# Lung Cancer Risk in Painters: A Meta-Analysis

**DOI:** 10.1289/ehp.0901402

**Published:** 2009-10-22

**Authors:** Neela Guha, Franco Merletti, Nelson Kyle Steenland, Andrea Altieri, Vincent Cogliano, Kurt Straif

**Affiliations:** 1 Section of IARC Monographs, International Agency for Research on Cancer, Lyon, France; 2 Unit of Cancer Epidemiology, Department of Biomedical Sciences and Human Oncology, University of Turin, Turin, Italy; 3 Department of Environmental and Occupational Health, Rollins School of Public Health, Emory University, Atlanta, Georgia, USA; 4 Emerging Risks Unit, European Food Safety Authority, Parma, Italy

**Keywords:** epidemiology, lung cancer, meta-analysis, painter

## Abstract

**Objective:**

We conducted a meta-analysis to quantitatively compare the association between occupation as a painter and the incidence or mortality from lung cancer.

**Data sources:**

PubMed and the reference lists of pertinent publications were searched and reviewed. For the meta-analysis, we used data from 47 independent cohort, record linkage, and case–control studies (from a total of 74 reports), including > 11,000 incident cases or deaths from lung cancer among painters.

**Data extraction:**

Three authors independently abstracted data and assessed study quality.

**Data synthesis:**

The summary relative risk (meta-RR, random effects) for lung cancer in painters was 1.35 [95% confidence interval (CI), 1.29–1.41; 47 studies] and 1.35 (95% CI, 1.21–1.51; 27 studies) after controlling for smoking. The relative risk was higher in never-smokers (meta-RR = 2.00; 95% CI, 1.09–3.67; 3 studies) and persisted when restricted to studies that adjusted for other occupational exposures (meta-RR = 1.57; 95% CI, 1.21–2.04; 5 studies). The results remained robust when stratified by study design, sex, and study location and are therefore unlikely due to chance or bias. Furthermore, exposure–response analyses suggested that the risk increased with duration of employment.

**Conclusion:**

These results support the conclusion that occupational exposures in painters are causally associated with the risk of lung cancer.

Lung cancer is the most common cancer diagnosis worldwide and is the major cause of cancer mortality, particularly among men. The International Agency for Research on Cancer (IARC) estimated that there were > 900,000 new cases of lung cancer each year among men and > 330,000 among women (IARC 2001, 2003). Approximately 90% of the lung cancer burden in developed countries is attributed to smoking, which acts either independently or synergistically with other occupational, lifestyle, or hereditary risk factors (Boffetta and Trichopoulos 2002; Peto et al. 1994). Several agents encountered in the occupational setting, such as asbestos, polycyclic aromatic hydrocarbons, arsenic, beryllium, cadmium, chromium(VI), and nickel compounds, are established carcinogens that target the lung (IARC 2008).

An increased incidence and mortality from lung cancer has been observed in painters, an occupation that employs several million people worldwide (IARC 1989). This has led IARC to classify occupational exposure as a painter as “carcinogenic to humans” (Group 1) (IARC 1989, in press; Straif et al. 2007). Painters are exposed to many known and suspected lung carcinogens through inhalation or dermal contact (IARC 1989; Siemiatycki et al. 2004), such as talc containing asbestos fibers, chromium VI compounds, chlorinated solvents, and cadmium compounds (IARC 1987, 1995, 1999, in press; Straif et al. 2009), although the specific causative agents have not yet been identified.

Cohort and record linkage studies demonstrating a relatively consistent increased incidence and mortality from lung cancer among painters [Alexander et al. 1996; Boice et al. 1999; Dubrow and Wegman 1984; Dunn and Weir 1965; Enterline and McKiever 1963; Gubéran et al. 1989; Guralnick 1963; Hrubec et al. 1995; Logan 1982; Menck and Henderson 1976; Office of Population Censuses and Surveys (OPCS) 1958, 1971, 1978, 1986, 1995; Petersen and Milham 1980; Pukkala 2009; van Loon et al. 1997; Whorton et al. 1983] have supported the IARC Group 1 classification, although potential confounding by tobacco smoking could not be ruled out in several of these studies. (Here we refer to record linkage studies as a subset of cohort studies where two databases are linked, such as a cohort of painters derived from census data and national mortality data, with only minimum demographic information available for the cohort.) Case–control studies have also shown that occupational exposure as a painter is a risk factor for lung cancer (Bethwaite et al. 1990; Bouchardy et al. 2002; Breslow et al. 1954; De Stefani et al. 1996; Finkelstein 1995; Milne et al. 1983; Pohlabeln et al. 2000; Wynder and Graham 1951), albeit somewhat less consistently (Baccarelli et al. 2005; Morabia et al. 1992; Muscat et al. 1998; Vineis et al. 1988; Wünsch-Filho et al. 1998), and the increased risk persisted after adjusting for the potential confounding by smoking (Brüske-Hohlfeld et al. 2000; Coggon et al. 1986; Decouflé et al. 1977; Houten et al. 1977; Jahn et al. 1999; Kjuus et al. 1986; Lerchen et al. 1987; Richiardi et al. 2004; Ronco et al. 1988; Viadana et al. 1976; Williams et al. 1977).

To assess the risk of lung cancer associated with occupational exposure as a painter, we conducted a meta-analysis of cohort, record linkage, and case–control studies to quantitatively compare the results of the different study designs and the potential confounding effect of smoking (by restricting to never-smokers), as well as other analyses to support the causal association. A thorough discussion of the individual studies included in the meta-analysis is not presented here but was summarized in the *IARC Monographs* (IARC 1989, in press). All of the studies reviewed, including the new studies published since the *IARC Monographs*, are summarized in Supplemental Material, Tables 1–3, available online (doi:10.1289/ehp.0901402.S1 via http://dx.doi.org/).

## Materials and Methods

### Selection criteria

All epidemiologic studies included in the previous *IARC Monographs* were considered (IARC 1989, in press). Further, we searched PubMed (National Center for Biotechnology Information 2009) for articles in any language describing lung cancer in painters referenced in or published since the previous *IARC Monograph* (IARC 1989) through 24 August 2009, using the following search terms [by text word (tw), MeSH heading (mh), or publication type (pt)]: “paint*[tw]” or “varnish*[tw]” or “lacquer*[tw]”; and “cancer” or “neoplasms[mh]”; and “case-control study[mesh]” or “cohort study[mesh]” or “meta-analysis[mh]” or “review[pt]” or “risk factors[mh]” or “neoplasms/epidemiology” or “neoplasms/etiology” or “neoplasms/CI” or “occupational diseases/etiology” or “occupational diseases/epidemiology” or “occupational diseases/CI” or “occupational diseases/MO” or “occupational exposure/adverse effects” or “death certificates[mh]” or “epidemiologic methods[mh]”; and “lung.” We identified 121 publications after restricting results to studies in humans. From the PubMed search, 69 studies were excluded because they were not epidemiologic studies, did not include original data (they were review articles), did not assess occupation as a painter, or lung cancer was not the outcome. The reference lists of pertinent publications were also reviewed to capture relevant data sources that may not have been identified with the search criteria.

The definition of painter varied between studies and often included other occupations exposed to paints such as plasterers, glaziers, wallpaper hangers, artists, decorators, French polishers, and aerographers [see Supplemental Material, Table 4 (doi:10.1289/ehp.0901402.S1) for definitions]. It is likely that paperhangers and other aforementioned occupations work in the same job environment as painters or may also paint; therefore, we considered this category as painters (Carstensen et al. 1988).

To be included in this meta-analysis, studies had to report estimates of the relative risk (RR), odds ratio (OR), standardized incidence ratio (SIR), standardized mortality ratio (SMR), proportionate mortality ratio (PMR), or proportional registration ratio with corresponding 95% confidence intervals (CIs) for ever-versus-never occupation as a painter or have provided enough information that allowed for their computation. For studies that did not report the ever-versus-never painter category, we estimated the risk estimates and 95% CIs for these categories. For studies that reported only point estimates without corresponding CIs, *p*-values, or standard errors, or did not report the distribution of data to allow for computation of relative risks and CIs (also for nonoverlapping populations), we made conservative assumptions to estimate RRs and 95% CIs from the data provided on a study-by-study basis. These conservative assumptions underestimated the relative risk (toward the null) and overestimated the width of the CI (i.e., by doubling the variance to approximate a 95% CI adjusted for multiple factors).

For example, overlapping lung cancer cases among African-American (black) men was identified by Morabia et al. (1992) and Muscat et al. (1998). We accounted for this population overlap by approximating the proportion of black male participants (cases and controls) based on distributions presented in other publications detailing this population, applying this proportion to the distribution presented by Morabia et al. (1992) (for black and whites combined) to determine the number of overlapping subjects, and subtracting the overlapping subjects from the distribution presented in Muscat et al. (1998).

Studies were excluded if estimation was impossible. In Supplemental Material, Tables 1–3 (doi:10.1289/ehp.0901402.S1), we use brackets to indicate the RRs and 95% CIs we calculated. For studies with overlapping populations, we included only the publication with the most complete study population. Further comments on study quality and any exclusions made are presented in detail in Supplemental Material, Tables 1–3. In total, we included in the meta-analysis 17 cohort and record linkage studies, 29 case–control studies, and 12 proportionate mortality analyses.

### Data abstraction

All articles were assessed independently by three reviewers (A.A., F.M., N.K.S.) who extracted data that included authors, publication date, country of origin, characteristics of the study population including sex, and any details on the definition of painters, incidence versus mortality, lung cancer histology, observed and expected cancer cases (for cohort and proportionate mortality studies), number of exposed cases and controls (for case–control studies), yes/no adjustment for smoking or other occupational carcinogens, relative risks with corresponding 95% CIs, and results on exposure–response [see Supplemental Material, Tables 1–3 (doi:10.1289/ehp.0901402.S1)]. If adjusted and unadjusted results were reported, the most valid point estimate (i.e., adjusted for smoking and other variables) was abstracted. Any discrepancies in data collection were resolved by two other reviewers (N.G., K.S.).

### Summary statistics calculated for inclusion in the meta-analysis

For cohort and record linkage studies, relative risk estimates (SIR and SMR) were computed by dividing the observed number of cases by the expected number, based on an external reference population. The corresponding 95% CIs were estimated using the PAMCOMP program (Taeger et al. 2000). If only subgroup results (e.g., by sex or duration of exposure) were reported, fixed-effects models were used to combine stratum- specific data into one summary estimate [see Supplemental Material, Tables 1 and 2 (doi:10.1289/ehp.0901402.S1)].

Wherever possible for the proportionate mortality studies, we used proportional cancer mortality ratios (calculating expected proportions of cancer deaths based on the proportion of cancer mortality in the reference population) in the analysis instead of PMRs as a more conservative approach, because proportional cancer mortality ratios provide a better risk estimate for specific cancer sites when the PMR for all cancer is artificially inflated by a deficit in other causes of death (Dalager et al. 1980) [see Supplemental Material, Table 3 (doi:10.1289/ehp.0901402.S1)]. If several cancer sites are associated with a particular occupation, the PMR can underestimate the RR.

Subgroup analyses were conducted by further restriction to studies with stronger methodologies, such as those studies that adjusted for smoking (Baccarelli et al. 2005; Brüske-Hohlfeld et al. 2000; Burns and Swanson 1991; De Stefani et al. 1996, 2005; Dunn and Weir 1965; Hrubec et al. 1995; Jahn et al. 1999; Kjuus et al. 1986; Lerchen et al. 1987; Levin et al. 1988; Matos et al. 2000; Morabia et al. 1992; Muscat et al. 1998; Notani et al. 1993; Pezzotto and Poletto 1999; Pohlabeln et al. 2000; Pronk et al. 2009; Richiardi et al. 2004; Ronco et al. 1988; Siemiatycki 1991; van Loon et al. 1997; Viadana et al. 1976; Vineis et al. 1988; Williams et al. 1977; Wünsch-Filho et al. 1998; Zahm et al. 1989; Zeka et al. 2006), other occupational risk factors (Jahn et al. 1999; Ronco et al. 1988; Stockwell and Matanoski 1985; van Loon et al. 1997), or population-based case–control studies that adjusted for smoking (Brüske-Hohlfeld et al. 2000; Burns and Swanson 1991; Coggon et al. 1986; Jahn et al. 1999; Lerchen et al. 1987; Levin et al. 1988; Pohlabeln et al. 2000; Richiardi et al. 2004; Ronco et al. 1988; Siemiatycki 1991; Vineis et al. 1988; Zahm et al. 1989; Zeka et al. 2006). Only four of the cohort and record linkage studies provided information on smoking status (Dunn and Weir 1965; Hrubec et al. 1995; Pronk et al. 2009; van Loon et al. 1997).

To allow for inclusion in the meta-analysis, we calculated 95% CIs if they were not presented in the original paper. If a 90% CI was presented and if the upper limit (UL) and lower limit (LL) were proportionally symmetric around the risk ratio (for RR and OR; i.e., if UL/RR = RR/LL), an estimate of the standard error (SE) was calculated by SE = (ln UL – ln LL/3.29), where 3.29 = 2 × 1.645 for 90% CIs. If only a *p-*value for the null hypothesis was presented, then a test-based SE was estimated using SE = (ln RR)/*Z_p_*, where *Z_p_* is the value of the standard-normal test statistic corresponding to the *p-*value using a two-tailed test. The UL and LL of the 95% CI were estimated by RR ± 1.96 (SE), where *Z_p_* = 1.96 if *p* = 0.05 using a two-tailed test (Rothman et al. 2008). A 95% CI corresponding to an unadjusted RR was used in the meta-analysis if a paper did not present enough data to allow for estimation of the adjusted CI.

### Statistical analysis

Because cancer incidence data are often more accurate than mortality data, we used SIRs in the analyses instead of SMRs whenever both were presented. However, mortality data for lung cancer are a very reasonable proxy for incidence because of the high fatality of lung cancer and the good quality of data from death certificates (Schottenfeld and Fraumeni 2006). We performed a separate meta-analysis for proportionate mortality studies. The PMRs were, however, not included in the overall meta-analyses because of their often lower quality of exposure assessment and their additional potential for bias. Assuming that the different effect estimates (e.g., SMR, SIR, RR, OR) represent the relative risk, the data were combined for all of the cohort, record linkage, and case–control studies. Subanalyses were also performed by stratifying on study design.

Many of the cohort and record linkage studies used an external reference population to calculate the expected cases. The use of an external reference population may result in a healthy worker effect, so that incidence or mortality rates of cancer in the exposed cohort may spuriously appear lower than in the general population. When the external reference rates used to calculate the expected cases are usually assumed to be known without error, an estimate of the exposure coefficient in a regression could be obtained by a weighted linear regression of the natural log of the adjusted SMR on exposure (Sutton et al. 2000). The risk estimates from nested case–control studies were included with the analysis of cohort studies because, essentially, this design can represent a more efficient way to analyze cohort studies and does not suffer from the problems associated with control selection in a case–control study. Summary ORs (meta-ORs) were obtained separately from the meta-analysis of case–control studies. Subgroup analyses were performed stratified by sex, study region, study design, types of adjustment, and duration of employment.

The *I*^2^ statistic quantifies the extent of inconsistency among the studies (Higgins and Thompson 2002). *I*^2^ values of 25–50% indicate moderate inconsistency, whereas values > 50% reflect large inconsistencies among studies. We present the *I*^2^ values instead of the Cochran’s *Q*-statistic because the *Q*-statistic informs about the presence or absence of heterogeneity but does not quantify the extent (Huedo-Medina et al. 2006). We used both random- and fixed-effect models, with weights equal to the inverse of the variance, to calculate a summary risk estimate (DerSimonian and Laird 1986). Results from random-effects models, which account for heterogeneity among studies, are presented.

We conducted sensitivity analyses by dropping one study at a time and examining its influence on the summary effect estimates. Forest plots were used to graphically display the data (Lewis and Clarke 2001). Publication bias was visually assessed using Funnel plots (Deeks et al. 2005). We performed all statistical analyses using STATA (version 10.0; StataCorp, College Station, TX, USA), employing the “metan” command for the meta-analyses (Bradburn 2004).

## Results

**Figure 1:**
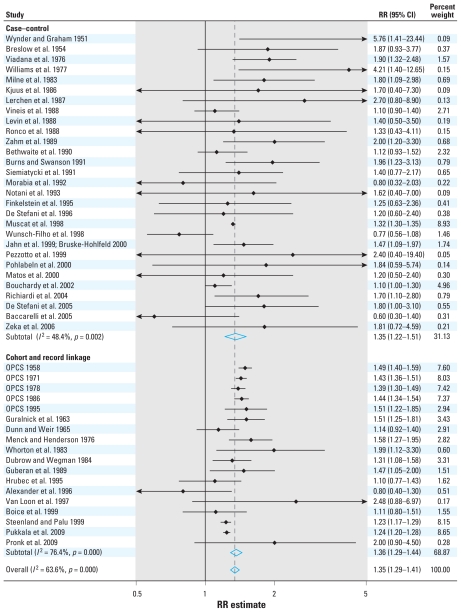
Meta-analysis of all studies assessing lung cancer among persons with occupation as a painter, stratified by study design. Weights are from random-effects analysis. The relative risk estimate for each study is represented by a black diamond, and the horizontal line shows the corresponding 95% CI. The dashed line marks the combined estimate, and the vertical solid line represents no association.

We reviewed 74 reports published since 1951 assessing the relationship between occupation as a painter and the risk of lung cancer [see Supplemental Material, Tables 1–3 (doi:10.1289/ehp.0901402.S1)]. The estimates of the relative risk reported in 47 independent studies ranged from 0.60 to 5.76, with 43 studies reporting an RR > 1.0 (Tables 1 and 2). The combined analysis of 18 cohort and record linkage studies (meta-RR = 1.36; 95% CI, 1.29–1.44; *I*^2^ = 76.4%, *p* = 0) and 29 case–control studies (meta-OR, 1.35; 95% CI, 1.22–1.51; *I*^2^ = 48.4%, *p* = 0.002), including > 11,000 incident cases and/or deaths from lung cancer among painters, demonstrated a significantly increased risk overall in persons who had ever reported occupation as a painter (meta-RR = 1.35; 95% CI, 1.29–1.41; *I*^2^ = 63.6%, *p* = 0) (Figure 1). Although the results of 13 proportionate mortality studies were not included in the combined analysis, they also demonstrated a significantly increased risk of lung cancer in painters (meta-PMR, 1.22; 95% CI, 1.17–1.28). The Forest plot (Figure 1) shows that there was no obvious trend in risk (at least no obvious trend toward a reduction in risk) over time. An influence analysis showed that dropping individual studies did not significantly alter the results (data not shown).

Relative risks were higher in female painters (meta-RR = 2.04; 95% CI, 1.59–2.62) (Jahn et al. 1999; Muscat et al. 1998; OPCS 1958, 1971; Pronk et al. 2009; Pukkala 2009; Zeka et al. 2006) than in males (meta-RR = 1.37; 95% CI, 1.29–1.44). Although there were only seven studies among female painters, the meta-RR was statistically significant. Stratification by study region showed that relative risks were highest in Asia (meta-RR = 1.71; 95% CI, 0.97–3.03; *I*^2^ = 0%, *p* = 0.86), similar in Europe (meta-RR = 1.38 95% CI, 1.28–1.48; *I*^2^ = 75.8%, *p* = 0) and North America (meta-RR = 1.35; 95% CI, 1.26–1.45; *I*^2^ = 56.4%, *p* = 0.001), and lower in South America (meta-RR = 1.17; 95% CI, 0.77–1.76; *I*^2^ = 48.8%, *p* = 0.10). Of the few studies that reported results for specific histologies (De Stefani et al. 1996, 2005; Pezzotto and Poletto 1999; Richiardi et al. 2004; Siemiatycki et al. 1987), relative risks were generally highest among those diagnosed with small-cell cancer, although the CIs were wide because of the small number of cases and because results for the different histologic entities were not reported consistently.

**Figure 2:**
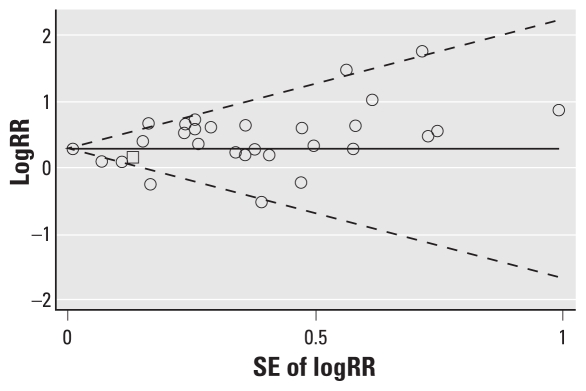
Begg’s funnel plot with pseudo-95% CIs to assess publication bias in case–control studies of lung cancer among persons reoporting occupation as a painter.

There appeared to be no evidence of publication bias among cohort and record linkage studies (data not shown). However, visual inspection of the funnel plot for 30 independent case–control studies demonstrated some evidence of publication bias: the plot was slightly skewed with a deficit of smaller nonpositive studies (represented by large SEs) (Figure 2). When restricting the analysis to the larger case–control studies that showed both positive and negative results, the meta-OR remained significantly elevated (meta-OR, 1.31; 95% CI, 1.18–1.45; *I*^2^ = 51.6%, *p* = 0.003). There was little difference in the results of case–control studies stratified by hospital-based controls (meta-OR, 1.37; 95% CI, 1.09–1.74; *I*^2^ = 59.3%, *p* = 0.002) or population-based controls (meta-OR, 1.34; 95% CI, 1.18–1.51; *I*^2^ = 25.9%, *p* = 0.16), although the population-based studies were less heterogeneous.

We performed additional analyses to examine the summary estimates when restricted to population-based case–control studies that adjusted for tobacco smoking or other occupational exposures. Restricting to population-based case–control studies that adjusted for smoking demonstrated less heterogeneity between studies and strengthened the results (meta-OR, 1.41; 95% CI, 1.23–1.61; *I*^2^ = 0%, *p* = 0.45). Four cohort studies reported smoking-adjusted results (Dunn and Weir 1965; Hrubec et al. 1995; Pronk et al. 2009; van Loon et al. 1997), with a meta-RR of 1.22 (95% CI, 0.97–1.52; *I*^2^ = 23.7%, *p* = 0.27), slightly lower than the meta-RR for cohort studies that did not adjust for smoking (meta-RR = 1.38; 95% CI, 1.30–1.46; *I*^2^ = 80.4%, *p* = 0). An analysis restricted to never-smokers (meta-RR = 2.00; 95% CI, 1.09–3.67; *I*^2^ = 0%, *p* = 0.97) (Kreuzer et al. 2001; Pronk et al. 2009; Zeka et al. 2006) and never-smokers and nonsmokers (meta-RR = 1.96; 95% CI, 1.15–3.35; *I*^2^ = 0%, *p* = 0.99) (Pohlabeln et al. 2000) demonstrated stronger associations than overall estimates. Regardless of study design, the studies that adjusted for other occupational exposures as well as smoking further strengthened the results (meta-RR = 1.57; 95% CI, 1.21–2.04; *I*^2^ = 0%, *p* = 0.68). Because estimates were relatively consistent between individual studies, regardless of study design, it is reasonable to assume that there is no important confounding by tobacco smoking or other occupational exposures among the studies that were not able to adjust for these factors.

Analysis by duration of exposure (< 10 years vs. ≥ 10 years, < 20 years vs. ≥ 20 years) (Baccarelli et al. 2005; Dalager et al. 1980; Levin et al. 1988; Pronk et al. 2009; Swanson et al. 1993) showed that those exposed ≥ 10 years (meta-RR = 1.95; 95% CI, 1.26–3.02; *I*^2^ = 0%, *p* = 0.63) or ≥ 20 years (meta-RR = 2.00; 95% CI, 1.01–3.92; *I*^2^ = 16.4%, *p* = 0.31) had a higher risk than those exposed < 10 years (meta-RR = 1.13; 95% CI, 0.77–1.65; *I*^2^ = 0%, *p* = 0.46) or < 20 years (meta-RR = 1.37; 95% CI, 0.89–2.13; *I*^2^ = 0%, *p* = 0.54) (reference category, 0 years of exposure), respectively.

## Discussion

Previous studies demonstrating an increased risk of lung cancer in painters have allowed IARC to classify occupation as a painter as carcinogenic to humans (Group 1) (IARC 1989, in press). This meta-analysis supports the IARC Group 1 classification by demonstrating a 35% increased risk of lung cancer in painters after adjusting for smoking (meta-RR = 1.35; 95% CI, 1.21–1.51; *I*^2^ = 41.2%, *p* = 0.01). This association was stronger for population-based case–control studies (meta-OR, 1.34; 95% CI, 1.18–1.51; *I*^2^ = 25.9%, *p* = 0.16) or studies that adjusted for other potentially confounding occupational exposures (meta-RR = 1.57; 95% CI, 1.21–2.04; *I*^2^ = 0%, *p* = 0.68). Furthermore, exposure–response analyses suggested that the risk increased with duration of employment. Although paint composition or the painting environment could have differed by major geographic region, the results did not vary much when stratified by region (North America, Europe, Asia, and South America). This is the first meta-analysis that demonstrates a relative increase in incidence/mortality from lung cancer in persons occupationally exposed as painters when restricted to never-smokers (and also nonsmokers), as well as demonstrating a statistically significant, positive duration–response relationship.

It is important to note that the interpretation of a meta-SMR (or meta-SIR) for the cohort and record linkage studies is difficult because different reference populations were used in each study for the calculation of expected cases or deaths (Rothman et al. 2008). Although the cohort studies of painters could assess possibly higher exposures from longer periods of follow-up, exposure assessment in many of the record linkage studies was often crude: Occupation as a painter was usually assessed at a single time point in a census and then linked to death registries. Although there can be relatively poor correspondence between occupation recorded on death certificates and in census records (Dubrow and Wegman 1984; Enterline and McKiever 1963; Guralnick 1963; OPCS 1971, 1978) and there is a chance of false-positive results due to multiple testing of occupations in record linkage studies, the SMRs were remarkably consistent between individual studies, generally ranging between 1.10 and 2.57. This also suggested that the significant results were not likely due to chance. Thus, the approach to combine the cohort and record linkage study SMRs for calculating a meta-SMR seemed to be justified.

In case–control studies, painters may only form a small proportion of the study population, but the full occupational history and additional information on lifestyle factors allowed several studies to adjust for tobacco smoking and some for other occupational carcinogens. An increased lung cancer risk associated with painting was consistently demonstrated in the case–control studies, suggesting that occupation as a painter is a risk factor for lung cancer. Population-based case–control studies may be less subject to selection biases than hospital-based case–control studies (Rothman et al. 2008) because there is generally no concern about the appropriate source population if indeed the general population is represented. However, if response rates are low in population controls, this could result in a lack of comparability with cases and therefore be prone to selection biases. A subanalysis comparing the meta-OR of hospital- based and population-based case–control studies showed similar results.

Estimates of the PMR may be biased if the population under study does not share the same distribution of mortality as the standard population used to compute the proportions for categories other than the ones studied (Rothman et al. 2008). However, the proportionate mortality analyses also showed significantly elevated relative risks for lung cancer in painters within the same range of effect as the analyses overall and in cohort studies, further suggesting that these results remained robust to these biases.

Smoking-adjusted estimates were available for 23 of 29 case–control studies and in only 4 of 18 cohort and record linkage studies. The robustness of the summary estimates after adjusting for tobacco use, and the higher relative risk in never-smokers, suggest that residual confounding by tobacco use is unlikely and that occupation as a painter is independently associated with the risk of lung cancer.

In women, the meta-RR was similar for all studies (meta-RR = 2.04; seven studies) (Jahn et al. 1999; Muscat et al. 1998; OPCS 1958, 1971; Pronk et al. 2009; Pukkala 2009; Zeka et al. 2006) and for studies restricted to never-smokers (meta-RR = 2.00; three studies) (Kreuzer et al. 2001; Pronk et al. 2009; Zeka et al. 2006), further strengthening the evidence that the results are not confounded by smoking. However, female painters (and never-smoking females) may not actually have a higher risk of lung cancer compared with male painters (meta-RR = 1.37; 39 studies). The relative risk in women is higher, which may be due to the fact that women have a lower background lung cancer risk than men (Schottenfeld and Fraumeni 2006).

The robustness of the results is also indicated by the presence of a duration–response relationship, with higher RRs seen for exposure over ≥ 10 years (meta-RR = 1.95) and ≥ 20 years (meta-RR = 2.00) compared with those with < 10 and < 20 years of exposure, respectively (the reference category was no exposure).

Some painters (e.g., in the construction industry) could have been exposed to asbestos. Indeed, a number of studies have shown an increased risk of mesothelioma in painters (Brown et al. 2002; Peto et al. 1995), which is most likely due to occupational asbestos exposure. However, taking into account that the exposure–response relationship for pleural mesothelioma is very different from that for lung cancer, potential asbestos exposure cannot explain all of the increase in lung cancer. Therefore, other suspected carcinogens to which painters are exposed, such as chlorinated solvents, chromium VI compounds, and cadmium compounds (IARC 1987, 1995, 1999, in press; Straif et al. 2009), may also partially explain the increased risk of lung cancer. Very few studies reported results for specific suspected causative agents. van Loon et al. (1997) reported a positive exposure–response relationship with paint dust and Siemiatycki et al. (1987) found a suggestive association with mineral spirits, whereas Alexander et al. (1996) did not find an increased risk of lung cancer in a cohort of painters and other employees in the aerospace industry exposed to chromium VI compounds.

## Conclusion

There is great variability and complexity in painting environments, which complicates the interpretation of epidemiologic studies of lung cancer risks in painters. Painters are exposed to a wide variety of chemical mixtures, with compositions that change over time. In more recent decades, a number of hazardous chemicals—including benzene, some other solvents, phthalates (plasticizers), and lead oxides—have been reduced or replaced in paint, although these chemicals are still used in some countries. This trend in reducing exposures to hazardous chemicals in paint has been promoted by the increasing use of water-based paints and powder coatings. New formulations may also contain lower-toxicity solvents, neutralizing agents (e.g., amines), and biocides (IARC 1989, in press). However, this has not yet resulted in lower relative risks for lung cancer in painters, as reported in the more recent observational epidemiologic studies. The elevated risk of lung cancer may also be partly due to the role that other substances may play in increasing the risk of lung cancer among painters.

Although there was not enough information in the studies provided to assess the association of lung cancer with specific chemical agents encountered in painting, the robustness of the estimates in the subgroup analyses (by sex, region, study design, and controlling for smoking and other occupational exposures) and the stronger associations seen in specific subgroups (by duration of exposure) support the conclusion that occupational exposures in painters are causally associated with the risk of lung cancer. Because several million people are employed as painters worldwide and because lung cancer is the most common cancer in painters, even a modest increase in the relative risk is remarkable. It is important for cancer control and prevention to design studies with better exposure assessment to identify the underlying carcinogenic agents encountered in painting.

## Figures and Tables

**Table 1:** Cohort and record linkage studies assessing lung and respiratory cancer among persons with occupation as a painter by publication date.

Reference, location, and time period	Cohort description	Exposure assessment	Exposure categories	No. of cases/deaths	HR/RR/SIR/SMR (95% CI)	Adjustment for potential confounders
Pronk et al. 2009, Shanghai, China 1996–2005	71,067 never-smoking women who held a job outside the home	Detailed lifetime occupational histories for each job held > 1 year from in-person interview	Painter (construction, automotive industry, and other users)	6	HR: 2.0 (0.9–4.5)	Passive smoking, family history of cancer, education
			Years employment^a^			
			< 10	1	0.83 (0.12–5.90)	Age, passive smoking (smokers excluded), education level, family history of lung cancer
			≥ 10	5	2.75 (1.12–6.73)	
			< 20	5	2.17 (0.89–5.31)	
			≥ 20	1	1.36 (0.19–9.75)	

Pukkala et al., in press, Denmark 1971–2003, Finland 1971–2005, Iceland 1982–2004, Norway 1961–2003, Sweden 1961–2005	15 million people in the 1960, 1970, 1980/1981, and/or 1990 censuses and the 2.8 million incident cancer cases diagnosed in these people in a follow-up until about 2005 were linked to Nordic national registries	Occupation from self-administered census questionnaire	Painters	[3,465]	SIR: [1.24 (1.20–1.28)]	Country, sex, age, period
			Men	3,418	1.23 (1.19–1.28)	
			Women	47	1.90 (1.40–2.53)	

Boice et al. 1999, Lockheed Martin Plant, Burbank, Los Angeles County, CA, USA 1960–1996	1,216 painters (1,139 men, 77 women) employed ≥ 1 year in the aircraft industry	Detailed job history from work history cards	Painter	41	SMR: 1.11 [0.80–1.51]	Age, sex, race, calendar year

Steenland and Palu 1999, California, Missouri, New York, Texas, USA, 1975–1994	42,170 painters and 14,316 nonpainters with ≥ 1 year union membership	Job titles inferred from union membership records that identified the members’ specialty affiliation and trade of the local union	Painter	1,746	SMR: 1.23 (1.17–1.29)	Age, calendar time

van Loon et al. 1997, The Netherlands 1986–1990	58,729 men, 55–69 years of age, were enrolled from the general Dutch population	Paint exposure from a self-administered questionnaire and case-by-case expert assessment	Paint dust exposure			Age, other occupational exposures, smoking habits, dietary intake of vitamin C, β-carotene, and retinol
			Any^b^	18	RR: [2.41 (1.07–5.44)]	
			Low	4	2.29 (0.61–8.63)	
			High	14	2.48 (0.88–6.97)	
			*p*-Value for trend		< 0.01	

Alexander et al. 1996, Seattle, WA, USA 1974–1994	2,429 chromate-exposed workers employed ≥ 6 months in the aerospace industry	Exposure to chromium (VI) was estimated from industrial hygiene measurements and work-history records	All workers	15	SIR: 0.8 (0.4–1.3)	Age, sex, race, calendar year

Hrubec et al. 1995, USA 1954–1980	1,178 painters assembled from a roster of approximately 300,000 white male veterans of World War I	Occupation and usual industry of employment from mailed questionnaire	Painters, construction, and maintenance	36	SMR: 1.1 [0.77–1.43]	Smoking, age, calendar time

Bethune et al. 1995; OPCS 1995, England and Wales, United Kingdom 1976–1989	Men from the 1971 and 1981 census cohorts who died between 1976 and 1989	Occupation from death certificates	Painters and decorators	NG	SMR: 1.51 (1.22–1.85)	Age, sex, calendar year

Gubéran et al. 1989, Switzerland 1971–1984	1,916 male painters from the 1970 Geneva census	Occupation from the 1970 census	Painters	40	SIR: 1.47 [1.05–2.00]	Age, sex, matrimonial status, calendar year

OPCS 1986, Scotland, England, and Wales, United Kingdom 1979–1980, 1982–1983	Men in Great Britain who died during 1979–1980 and 1982–1983; mortality of men 15–74 years of age in England and Wales in 1981	Last full-time occupation from death certificate	Painters, decorators, French polishers Men	779	SMR: 1.44 [1.34–1.54]	Age, sex

Dubrow and Wegman 1984, Massachusetts, USA 1971–1973	34,879 white men > 20 years of age	Usual occupation from death certificate	Painters grouped	110	SMR : 1.31 [1.08–1.58]	Age

Whorton et al. 1983, San Francisco/Oakland SMSA, CA, USA 1976–1978	2,200 painting union members (2,197 men, 3 women)	1976–1977 union membership files	Painter	15	SIR: 1.99 [1.12–3.30]	Age, sex, year

OPCS 1978, England and Wales, United Kingdom 1970–1972	Registered deaths of 273,129 men	Last occupation recorded on the death certificate	Painters and decorators	847	SMR: 1.39 [1.30–1.49]	Age, sex

Menck and Henderson 1976, Los Angeles County, CA, USA 1968–1970	Pooled mortality and morbidity data of 2,161 deaths from lung cancer and 1,777 incident cases of lung cancer among white males	Last occupation from death certificates and surveillance registry files	Painter	87	SMR: 1.58 [1.27–1.95]	Age

OPCS 1971, England and Wales, United Kingdom 1959–1963	Registered deaths of men and women in England and Wales	Last occupation from death certificate	Painters and decorators			Age, sex
			15–64 years of age			
			Men and women	1,506	SMR: 1.43 [1.36–1.51]	
			Men	1,502	1.43 [1.36–1.50]	
			Single women	4	4.00 [1.09–10.24]	

Dunn and Weir 1965, California, USA 1954–1962	Prospective study of > 68,000 men working in “suspicious” occupations (12,512 painters and decorators)	Men were enrolled based on their occupation, identified through unions, and mailed questionnaire	Painters and decorators	91	SMR: 1.14 [0.92–1.40]	Age, smoking

[Bibr b23-ehp-118-303][Bibr b26-ehp-118-303], USA 1950	Men who died in the USA in 1950	Usual occupation and industry recorded from death certificates	Painters and plasterers	118	SMR: 1.51 [1.25–1.81]	Age, race

OPCS 1958, England and Wales, United Kingdom 1949–1953	Registered deaths of 221,941 men and women in the broad occupational category of painters and decorators	Occupation at time of death or last occupation from death certificates	Other painters and decorators			Age, sex
			Men and women	912	SMR: [1.49 (1.40–1.59)]	
			Men	909	[1.49 (1.40–1.59)]	
			Single women	3	3.00 [0.62–8.77]	

Abbreviations: HR, hazard ratio; NG, not given; SMSA, standard metropolitan statistical area. Values in brackets were calculated by us.

a Information obtained by contacting authors.

b Calculated using a fixed-effects model.

**Table 2:** Case–control studies of the association between lung cancer and occupation as a painter by publication date.

Reference, location, and time period	Characteristics of cases	Characteristics of controls	Exposure assessment	Exposure	No. of exposed cases	OR (95% CI)	Adjustment for potential confounders
Zeka et al. 2006, Czech Republic, Hungary, Poland, Romania, Russia, Slovakia, United Kingdom 1998–2002	223 never-smoking cases (48 men, 175 women)	1,039 nonsmoking controls (534 men, 505 women)	Lifetime occupational histories for jobs held ≥ 1 year from in-person interview	Painters			
				Men and women	6	[1.81 (0.72–4.59)]	None
				Women	6	1.8 (0.53–6.0)	Sex, age, study center

Baccarelli et al. 2005, Leningrad Province (Russia) 1993–1998	540 (474 men, 66 women)	582 (453 men, 129 women) individuals with autopsy-based diagnoses of non–cancer-related and non–tobacco-related conditions, frequency matched by sex, age, area, year of death	Lifetime occupational histories from personal records	Ever painters	10	0.6 (0.3–1.4)	Age, sex, smoking
				< 10 years	6	0.5 (0.2–1.5)	
				≥ 10 years	4	0.8 (0.2–3.0)	

De Stefani et al. 2005, Montevideo, Uruguay 1994–2000	338 men	1,014 males hospitalized for conditions not related to tobacco smoking, matched by age, residence and urban/rural status	Lifetime occupational history from in-person interview	Ever painter	26	1.8 (1.0–3.1)	Age, residence, urban/rural status, education, smoking status and years since quitting and age at start, no. of cigarettes per day
				Employment (years)			
				1–20		9.6 (2.6–36.0)	
				≥ 21		1.2 (0.6–2.2)	
				*p* for trend		0.07	

Richiardi et al. 2004, Turin and Eastern Veneto, Italy, 1990–1992	956 men	1,253 male population-based controls, matched by study area, 5-year age groups	Lifetime occupational history from in-person interview	Ever painters	62	1.7 (1.1–2.8)	Age, study area, smoking (never, ex-, active smokers), no. of job periods, education

Bouchardy et al. 2002, cantons of Basel, Geneva, St Gall, Vaud, and Zurich, Switzerland, 1980–1993	9,106 men	49,028 male non–lung cancer registrants	Longest, current, or most recent occupation as recorded at the time of registration (main or best-specified occupation in Zurich Registry)	Plasterers and painters (in the construction industry)	273	1.1 (1.0–1.3)	Age, registry, civil status, period of diagnosis, nationality, urban/rural residence, socioeconomic status, histologic confirmation, information from death certificate only (cases)

Matos et al. 2000, Buenos Aires, Argentina, 1994–1996	200 men	397 male controls hospitalized for non–tobacco-related conditions, matched by hospital and age	Full occupational history from in-person interview. Further details requested for occupations held > 1 year.	Ever painters	16	1.2 (0.5–2.4)	Age, hospital, smoking (pack-years), other occupations with significant ORs (*p* < 0.05)

Pohlabeln et al. 2000, 12 centers in Germany, Italy, Portugal, Sweden, United Kingdom, France, and Spain, 1988–1994	650 nonsmoking cases^a^ (509 women, 141 men)	1,542 nonsmoking controls (1,011 females, 531 males)	In-person interview for lifetime occupational history	Ever painters (men)	6	1.84 (0.59–5.74)	Age, center

Jahn et al. 1999; Bruske-Hohlfeld et al. 2000, bGermany, 1988–1993, 1990–1996	686 women, 3,498 men	712 female and 3,541 male population controls	Full occupational history and supplementary job-specific modules from in-person interview	Ever painters (women)	13	3.0 (0.73–12.33)	Smoking, asbestos, education, age, region of residence
				Ever painters/lacquerers			
				Men	147	1.42 (1.05–1.92)	
				Men and women	[160]	[1.47 (1.09–1.97)]^c^	

Pezzotto and Poletto 1999, Rosario City, Argentina, 1992–1998	367 men	586 hospital-based males controls admitted for a non–smoking-related disease at the same hospitals for traumatic conditions, urologic diseases, acute surgical conditions, and other illnesses, matched by age (± 3 years); mean age 60.1 ± 10.2 years	Lifetime occupational history for each job held > 1 year from standardized questionnaire	House painters	4	2.4 (0.4–19.4)	Age, smoking habit, lifelong cigarette consumption

Muscat et al. 1998, New York City, Long Island, NY; Philadelphia, PA; Washington, DC; Detroit, MI; Chicago, IL, USA, 1978–1996	365 black men and 185 black women	251 male and 135 female black patients; conditions unrelated to tobacco use, matched by race, sex, 5-year age groups, month of diagnosis	Only “usual” occupation and whether the job entailed regular exposure to an occupational exposure (for a minimum of 8 hr/week) was obtained from interviews with subjects or their next of kin or death certificates	Ever painters	[24]	[1.32 (1.30–1.35)]^c^	Age, education, smoking
				Men^d^	[19]	[0.68 (0.29–1.59)]	
				Women	5	1.8 (0.3–12.3)	

Wünsch-Filho et al. 1998, São Paulo, Brazil, 1990–1991	398 cases (307 men, 91 women)	860 controls (546 men, 314 women) hospitalized for non–tobacco-related conditions, matched by age, sex, hospital	Full occupational history from in-person interview	Ever painters (men)	128	0.77 (0.56–1.08)	Age, sex, hospital, smoking, cancer in family, migration history, socioeconomic status

De Stefani et al. 1996, Montevideo, Uruguay, 1993–1994, South America	270 men	383 male hospital-based controls: other cancer sites except oral cavity, pharynx, esophagus, stomach, larynx, and bladder	Lifetime occupational history from in-person interview	Ever painters	18	1.2 (0.6–2.4)	Age, residence, education, tobacco smoking (pack–years), alcohol consumption
				Employment (years)			
				1–20		0.9 (0.2–3.0)	
				≥ 21		1.4 (0.6–3.1)	

Finkelstein 1995, Hamilton and Sault Ste-Marie, Ontario, Canada, 1979–1988	967 men	2,821 men who died of any cause other than lung cancer, matched by age, year of death, and city of residence	Occupation (job and industry) from death certificate	Painters and plasterers	16	1.25 (0.63–2.36)	Age, year of death, city of residence

Notani et al. 1993 Bombay, India, 1986–1990	246 men	212 male hospital-based controls diagnosed with cancers of the mouth and oro- or hypopharynx and noncancerous oral disease, frequency matched by age and community	Lifetime occupational history from in-person interview	Ever painters	6	1.62 (0.4–7.0)	Age, community, smoking (two groups)

Swanson et al. 1993, Detroit, MI, metropolitan area, USA,1984–1987	3,792 males (2,866 white, 926 black)	1,966 males (1,596 white, 370 black) with colon and rectal cancer	Lifetime occupational and smoking history from telephone interviews with subjects or their surrogates	Painting machine operators, black and white			Age at diagnosis, pack-years of cigarette smoking
				< 10 years	40	[1.19 (0.61–2.34)]^e^	
				≥ 10 years	40	[2.23 (1.05–4.73)]^e^	
				< 20 years	53	[1.15 (0.65–2.04)]^e^	
				≥ 20 years	27	[4.62 (1.61–13.31)]^e^	

Morabia et al. 1992, Detroit, MI; Chicago, IL; Philadelphia, PA; Pittsburgh, PA; New York, NY; Long Island, NY; San Francisco, CA; Birmingham, AL, USA, 1980–1989, American Health Foundation study	1,793 men	3,228 controls not hospitalized for lung cancer but including tobacco-related conditions; matched by age, race, hospital, smoking history, admission date	“Usual” occupation and exposure circumstances from in-person interview	Painters	[13]	0.8 (0.32–2.03)	Age, geographic area, race, smoking, study period

Burns and Swanson 1991, Detroit, MI, metropolitan area, USA	5,935 (3,918 males, 2,017 females)	3,956 (1,981 males, 1,975 females) with colon and rectal cancer	Lifetime occupational history from telephone interviews to the subjects or to their surrogates	Painters (usual occupation, grouped)	97	1.96 (1.23–3.13)	Age at diagnosis, race, smoking, sex

Siemiatycki 1991, Montreal, Canada, 1979–1985	857 men	533 population controls, 1,360 cancer controls	Lifetime occupational history from interview	Construction painter			Age, family income, ethnicity, respondent type, cigarette and alcohol index
				Any exposure	26	1.4 (0.77–2.17)	

Bethwaite et al. 1990, New Zealand, 1980–1984	4,224 men	15,680 male non–lung cancer registrants	Current/most recent occupation as recorded at the time of registration; smoking history obtained through telephone interview	Painter decorators, steel and other construction painters, car painters, spray painters, signwriters, other unclassified painters	88	1.12 (0.93–1.52)	Age

Zahm et al. 1989, Missouri, USA, 1980–1985	4,431 white male cases	11,326 white male non–lung cancer registrants	Occupation at the time of diagnosis abstracted from medical records	Painters, paper hangers, plasterers	37	2.0 (1.2–3.3)	Age, smoking

Levin et al. 1988, China, 1984–1985	733 men	760 age-matched population controls	Lifetime occupational history from interview	Ever painter	15	1.4 (0.5–3.5)	Age, smoking
				Duration (years)			
				< 10	7	1.9 (0.36–16.60)^f^	
				10–19	2	2.8 (0.07–62.47)^f^	
				20–29	5	2.2 (0.26–26.67)^f^	
				≥ 30	1	0.3 (0.01–5.81)^f^	
				> 10	8	[1.34 (0.26–6.92)]^e^	
				< 20	9	[2.35(0.44–12.47)]^e^	
				> 20	6	[1.18 (0.18–7.64)]^e^	

Ronco et al. 1988, Italy, 1976–1980	126 men	384 men who died from causes other than from smoking-related or chronic lung diseases	Lifetime occupational history from interview with next of kin	Painter	5	1.33 (0.43–4.11)	Age, year of death, smoking, other employment in suspect high-risk occupations

Vineis et al. 1988, Analysis of five case–control studies in Louisiana, Florida, Pennsylvania, Virginia, and New Jersey, USA, 1970s and 1980s	2,973 men	3,210 men	Lifetime occupational history from interview with subjects or next of kin	Painters	201	1.1 (0.9–1.4)	Age, birth cohort, smoking

Lerchen et al. 1987, New Mexico, USA, 1980–1982	771 cases (333 men, 173 women)	771 controls (499 men, 272 women)	Lifetime occupational history from interview	Ever construction painters (men)	9	2.7 (0.8–8.9)	Age, ethnicity, smoking

Coggon et al. 1986,^g^ Cleveland, Humberside, Cheshire counties, United Kingdom, 1975–1980	738 male bronchial cancer cases	1,221 other cancers	Occupation from mailed questionnaire	Painters and decorators	20	1.3 (0.62–2.72)	Age, smoking, residence, respondent

Kjuus et al. 1986, Norway, 1979–1983	176 men	176 age-matched hospital controls excluding those with physical or mental handicaps, poor general health, or diagnosed with chronic obstructive lung disease	Longest job held from interview and work site records	Painting, paper-hanging (occupation)	5	1.7 (0.4–7.3)	Age, smoking

Milne et al. 1983, Alameda County, CA, USA, 1958–1962	925 lung cancer deaths (747 men, 178 women)	4,880 deaths from other cancers (except pancreatic, bladder, nasal, kidney, hematopoietic) that are not known to be strongly associated with occupational risk factors (reported as the “reduced control group”)	Occupation from death certificates	Painters (men)	24	1.80 (1.09–2.98)^h^	Age

Williams et al. 1977, Atlanta, GA; Birmingham, AL; Colorado; Dallas-Ft. Worth, TX; Detroit, MI; Minneapolis-St. Paul, MN; Pittsburgh, PA; San Franciso–Oakland, CA, USA, Third National Cancer Survey	432 cases	2,173 patients with cancers other than lung, larynx, oral cavity, esophagus, bladder	Main lifetime employment from survey questionnaire	Painting (men)	12	4.21 (1.40–12.65) (*p* < 0.01)	Age, race, education, tobacco, alcohol, geographic location

[Bibr b80-ehp-118-303][Bibr b18-ehp-118-303][Bibr b28-ehp-118-303], Buffalo, NY, USA, 1956–1965	Lung cancer cases from 11,591 white male cancer cases	Noncancer admissions from the same cancer treatment center	Lifetime occupation from interview before diagnosis	Painter		
				Ever	42	1.90 (1.32–2.48)	Smoking, age

Breslow et al.1954, California, USA, 1949–1952	518 patients	518 hospital controls matched by hospital, age, sex, race	Interview	Construction and maintenance painters for ≥ 5 years	22	[1.87 (0.93–3.77)]	Hospital, age, sex, race

Wynder and Graham 1951, St. Louis, MO, USA, NG	200 cases	200 controls with a chest disease other than lung cancer	Lifetime occupational history from interview	Painter ≥ 5 years within the last 40 years	11	[5.76 (1.41–23.44)]	None

NG, not given. Values in brackets were calculated by us.

a Nonsmokers, subjects who smoked < 400 cigarettes during their lifetime.

b BIPS study in Bremen area and Frankfurt/Main area; GSF study in Nordrhein-Westfalen, Rheinland-Pfalz and Bayern, Saarland, Thuringen, and Sachsen.

c Fixed-effects model used to calculate a weighted average.

d The study partially overlaps with Morabia et al. 1992 and thus some estimations were used to eliminate the overlap in men and the estimated variance was doubled to approximate an adjusted CI.

e Calculated using a fixed-effects model.

f Variance was doubled to approximate an adjusted 95% CI.

g Included in the analysis restricted to case–control studies but excluded from the combined meta-analysis because of possible overlap with OPCS 1986.

h The CI was estimated by applying the ratio of reduced/total controls to the observed cell counts reported for the total control group.
